# “Education with art”: diabetes education through theater

**DOI:** 10.1186/1758-5996-7-S1-A188

**Published:** 2015-11-11

**Authors:** Claudia Mauricio Pieper, Sandra Maria Costa, Anna Wiltgen, Andrea Martins, Rosane Kupfer

**Affiliations:** 1 Instituto Estadual de Diabetes e Endocrinologia-IEDE, Rio de Janeiro, Brazil

## Background

How can we best engage the age group of 10 to 17 year olds to increase their awareness and knowledge of diabetes? Trying to solve this challenge, we started this project in a public hospital 5 yrs. ago in an unprecedented manner.

## Objectives

To improve adhesion of preteens and teens to the treatment of diabetes using theater techniques.

## Materials and methods

Longitudinal study, with the participation of 24 young people with diabetes type 1 (DM1) between 10-17 yrs. old. The acting classes are biweekly lasting, for 3 hrs., aiming to improve the expressions of feelings and at the same time the body language, as a way of working possible psychological barriers in the adherence to diabetes treatment. The program covers topics on diabetes (based on a Education Program) that are discussed through games and activities to increase the interest and reduce stressful situations. At the same time, is carried out a support group for parents with a psychologist. At the end of a year the group presented a play whose theme was based on the comics Uncle Julian. A simple and fun language provides a method of learning about various topics on diabetes in a playful manner.

## Results

The mean age of participants is 14.5 yrs. history of diabetes of 6.5 yrs. Glycated hemoglobin levels were measured (HbA1c) before and after each 3 or 4 months with an average drop of 1.2% in the group after 12 months. Among the subjects, “diet” was one of the most discussed.

## Conclusions

This challenging and unique project that uses theater as a form of diabetes education has achieved its main objective in relation to a better glycemic control and adherence to treatment in the group of young people with DM1.

